# New insights into the causes of human illness due to consumption of azaspiracid contaminated shellfish

**DOI:** 10.1038/srep09818

**Published:** 2015-04-30

**Authors:** O. P. Chevallier, S. F. Graham, E. Alonso, C. Duffy, J. Silke, K. Campbell, L. M. Botana, C. T. Elliott

**Affiliations:** 1 Advanced Asset Technology Centre, Institute for Global Food Security, Queen's University Belfast, Stranmillis Road, Belfast, BT9 5AG, UK; 2 Beaumont Research Institute, 3811 W Thirteen Mile Road, Royal Oak, MI, 48073; 3 Marine Institute, Rinville, Oranmore, Co. Galway, Ireland; 4 Department of Pharmacology, Faculty of Veterinary, Campus Lugo, USC, 27002 Lugo, Spain

## Abstract

Azaspiracid (AZA) poisoning was unknown until 1995 when shellfish harvested in Ireland caused illness manifesting by vomiting and diarrhoea. Further *in vivo/vitro* studies showed neurotoxicity linked with AZA exposure. However, the biological target of the toxin which will help explain such potent neurological activity is still unknown. A region of Irish coastline was selected and shellfish were sampled and tested for AZA using mass spectrometry. An outbreak was identified in 2010 and samples collected before and after the contamination episode were compared for their metabolite profile using high resolution mass spectrometry. Twenty eight ions were identified at higher concentration in the contaminated samples. Stringent bioinformatic analysis revealed putative identifications for seven compounds including, glutarylcarnitine, a glutaric acid metabolite. Glutaric acid, the parent compound linked with human neurological manifestations was subjected to toxicological investigations but was found to have no specific effect on the sodium channel (as was the case with AZA). However in combination, glutaric acid (1mM) and azaspiracid (50nM) inhibited the activity of the sodium channel by over 50%. Glutaric acid was subsequently detected in all shellfish employed in the study. For the first time a viable mechanism for how AZA manifests itself as a toxin is presented.

The first evidence of a previously unreported toxic threat from eating shellfish appeared in 1995 when a group of eight Dutch consumers suffered from food poisoning after consuming shellfish harvested around the shores of Ireland^1^. The symptoms described appeared to match those associated with classic diarrhoeic shellfish poisoning (DSP). However when the samples were analysed very low amounts of DSP toxins were found to be present, and the dinoflagellate organisms known to produce DSP toxins were not observed in the region of Ireland where the shellfish had been harvested. Another confounding piece of information came from a study whereby a group of mice were injected with shellfish extracts from the contaminated batch. These mice suffered from spasms in the hind legs, a slowly progressing paralysis and a range of neurotoxin-like symptoms^2^, never before observed with DSP contaminated shellfish^3^. Within a short period of time the structure of a ‘new’ marine biotoxin had been proposed^4^ and later revised^5^. This novel, complex polyether compound was named azaspiracid and its occurrence in Irish waters has been widely reported causing massive economic damage to the shellfish industry. Since its discovery over 150 papers have been published on azaspiracid dealing with factors such as the organism responsible for producing the toxin, namely *Azadinium*^6^ and *Amphidoma*^7^, the widespread geographical locations where the toxin has been found^8^,^9^,^10^, methods of analysis^11^,^12^ and identification of multiple azaspiracid analogues with differing toxicities^13^,^14^. However the determination of the mode of action of azaspiracid has remained elusive to a wide range of toxicologists who have attempted to determine its biological target^13^,^15^. One recent study claimed that the biological target had been identified as the hERG potassium ion channel^16^. However the 50% inhibitory concentrations of toxin required to inhibit K+ current were high (0.64–0.84µM) and not realistic with regards to concentrations found in contaminated shellfish (0.24µg AZA equivalents.g^−1^)^17^, causing intoxications at high doses^18^, and its mechanism of action is probably linked to apoptotic targets that respond with irreversible kinetics^19^, and at low doses^20^. Interestingly a study by Furey et al.^21^, observed that in animals injected with partially purified toxins from contaminated shellfish the effects were more severe than that induced by the pure compound. This group hypothesized that the presence of other azaspiracid analogues and/or matrix components could be responsible for these differences. Additionally severe cytotoxicity effects, not usually associated with marine toxins, were first observed by Flanagan et al.^22^, using crude shellfish extracts rather than pure toxin. A large project (ASTOX)^23^ focusing on the possible mechanisms of azaspiracid toxicity concluded that the presence of other DSP toxins (okadaic acid and yessotoxins) did not cause synergistic effects^24^. In addition they reported that when pigs were exposed to relatively high amounts of pure azaspiracid they did not show any symptoms associated with human intoxication. Based on existing evidence associated with azaspiracid's toxicity, the present authors hypothesized that there may be another compound (as yet unidentified) present in contaminated shellfish which acts in synergy with azaspiracid to cause the combination of toxic effects noted in those animals and humans exposed to contaminated shellfish. In attempt to prove this hypothesis correct, a complex and elaborate set of investigations were organised.

## Results

### Targeted analysis

**Figure 1:**
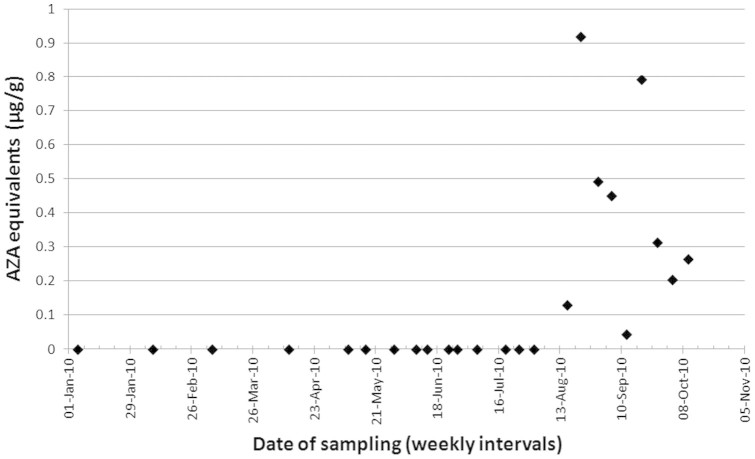
Concentration of azaspiracid (AZA equivalents ug.g^−1^) as determined by LC-MS/MS in mussel samples from Poulnaclough Bay, Co. Galway analysed from January to October 2010.

A unique set of blue mussels (*Mytilus edulis*) (n = 24), were collected from Poulnaclough Bay, Ballyvaghan Bay Co. Galway, Ireland between January and October 2010. The LC-MS/MS analysis of the shellfish samples (Fig. 1), through the Irish toxin monitoring programme, showed that between January 6^th^ and August 1^st^ (n = 15) no detectable amounts of azaspiracid toxins were present. However immediately after this a toxic episode was recorded whereby all samples between August 16^th^ and October 9^th^ (n = 9) had measureable amounts of toxin present. The peak contamination concentration on August 22^nd^ was 0.92 AZA equivalents (µg.g^−1^) which is approximately six fold greater than the limit set by the EU which has been established at 0.16 µg.g^−1^ (EU 853/2004). All toxin negative samples were pooled as were the toxin containing samples and from here on described as Pool 1 and Pool 2 respectively.

The importance of the presence of glutaric acid in shellfish in this study will become apparent in later sections. However as there are no previous reports on the presence of glutaric acid being measured in shellfish samples, a dedicated LC-MS/MS assay had to be developed. A small survey comprising of shellfish samples from the study and samples from commercial sources (n = 10) was then performed in order to estimate the glutaric acid presence and determine its concentration. Relatively high levels were detected ranging from 29.5 to 81.1 μg.g^−1^ of dry extract corresponding to 2.95 to 8.11 μg.g^−1^ of mussel (assuming that the average water content of the shellfish samples was 90%) (Table 1). No correlation between the levels of glutaric acid found in AZA contaminated (Pool 2) or non-contaminated (Pool 1) was observed.

### Untargeted analysis

**Figure 2:**
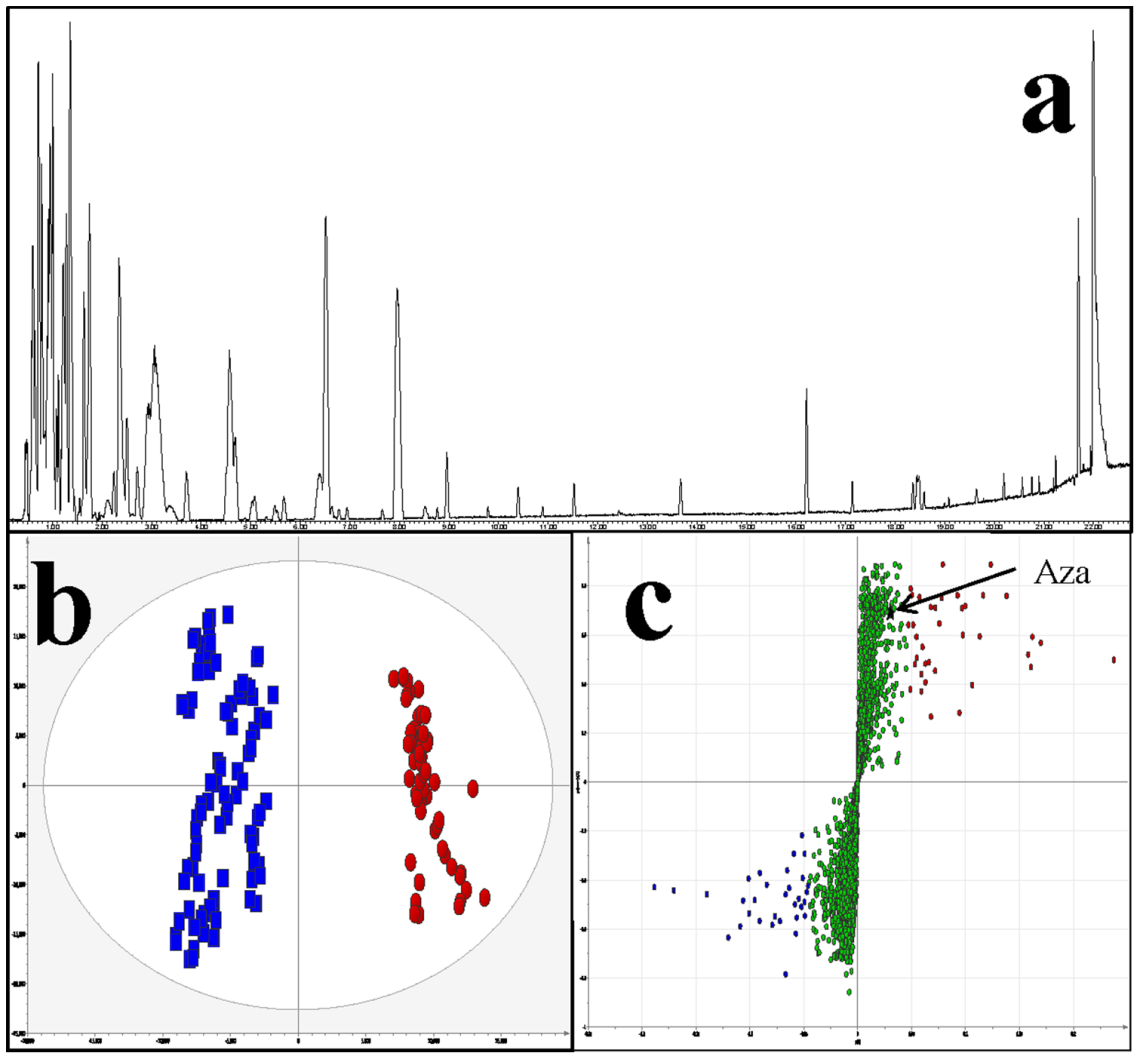
(a) Representative UPLC ESI+ chromatogram of the polar extract of mussel samples. (b) OPLS-DA scores plot representing AZA non-contaminated controls (blue squares; n = 92) vs. azaspiracid (AZA) contaminated samples (red circles; n = 56). Explained variance (R2) was 98.7%, the predictive ability (Q2) was 98.3% and RMSECV was 7.56%. (c) The s-plot which corresponds to Fig. 1c. Highlighted in red represent features which are at significantly higher concentrations in AZA samples when compared with controls. Highlighted in blue are features which are at significantly lower concentrations in AZA samples when compared with controls (non-contaminated mussel specimens).

**Figure 3:**
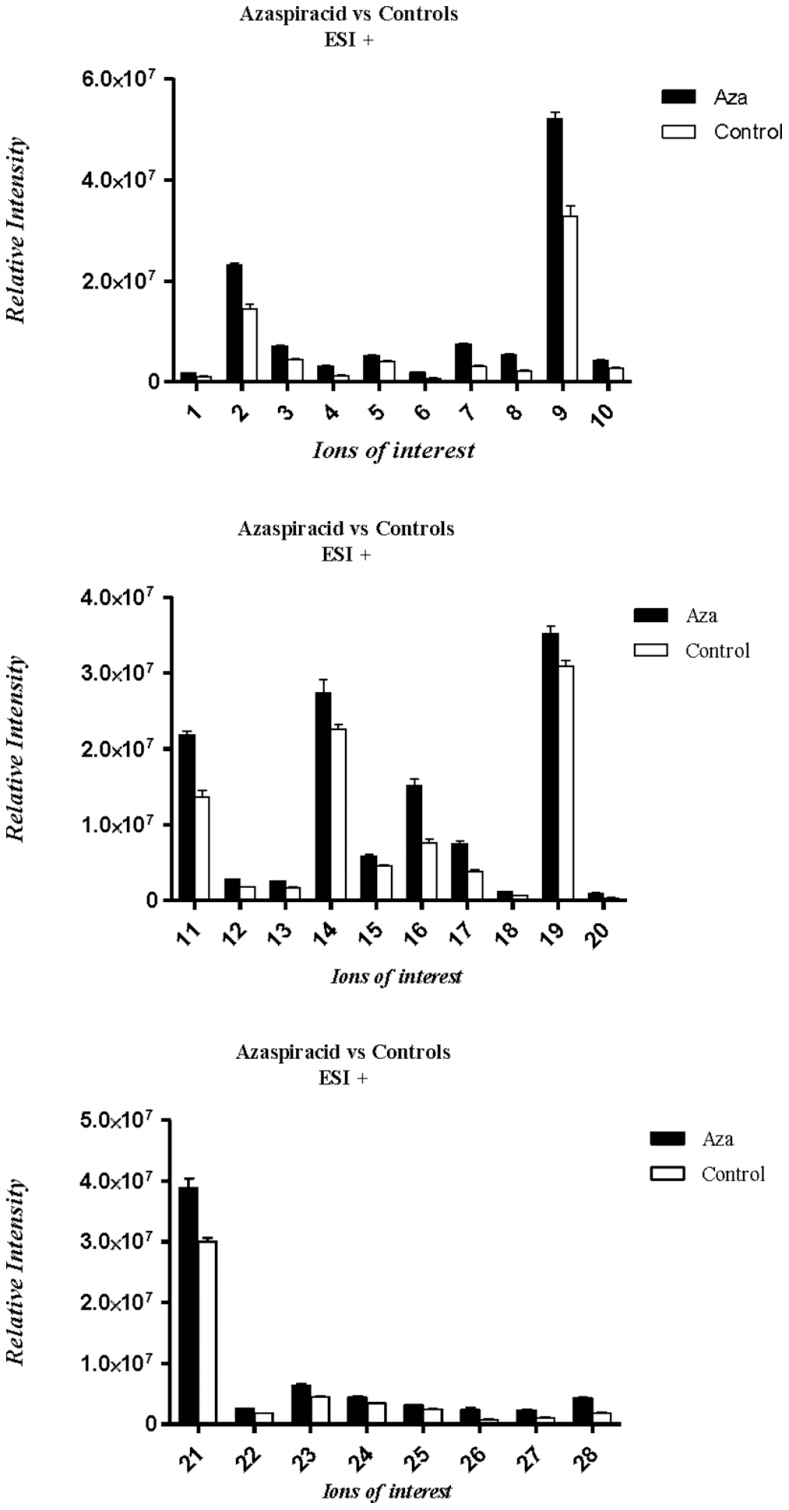
Graphical representation of the metabolites and their relative abundances in both AZA and control samples measured in ESI+. (Ions of interest which are found to be at higher abundances in AZA samples. Numbered columns relate to the features labelled in Table 2).

From a total of 4321 ions which were assigned by TransOmics v1.0 Software, 28 proved to be at significantly higher levels in AZA contaminated samples (Pool 2). These ions of interest (highlighted by the S-plot, Fig. 2c) at different levels between the two sample groups were analysed using a two tailed homoscedastic student t-test (assuming that the two sample groups were of equal variance) using Microsoft Excel (2010)^25^. Graphical representations of the data were produced using Prism v5.0 (Fig. 3). Using mass accuracy and isotope similarity as a filter, seven putative IDs for the features higher in AZA contaminated samples were identified (Table 2). Interestingly, azaspiracid was detected only in Pool 2 as expected but proved to be less statistically significant than the selected “ions of interest”. The compounds identified in the AZA contaminated samples were then reviewed with the aim of identifying a substance or substances known to be harmful to humans. Of these compounds glutarylcarnitine proved to be the most interesting by far. This compound is the main metabolite of glutaric acid and has been reported to have been identified in urine, plasma and CSF of patients with glutaric aciduria type I^26^. This autosomal recessive inborn error is caused by a severe deficiency of glutaryl-CoA dehydrogenase activity, which leads to the abnormal metabolism of lysine, hydroxylysine and tryptophan amino acids resulting in an accumulation of glutaric acid, 3-hydroxyglutaric acid and glutarylcarnitine^26^. This disease is characterized by acute neurological compromise with generalized convulsions, loss of motor skills and dystonia^27^,^28^. On the basis of the significant (p <0.001) increase in levels of the metabolite glutarylcarnitine in AZA contaminated samples, coupled with the well documented link with neurological disorders such as glutaric aciduria, glutaric acid was selected for further toxicology studies.

### Toxicology studies

#### The effects of glutaric acid and glutarylcarnitine on cellular viability and in hNav1.6 currents

Serial dilutions of glutaric acid ranging from 0.0001 to 100 mM were added to the culture medium of the neural cell line SH-SY5Y. After 24 hours or 1 hour incubation, a MTT test was performed to measure mitochondrial function as a marker of cellular viability. When glutaric acid was added directly to the cellular medium a decrease of cellular viability was produced, however the decrease in cellular viability observed was found to be due to the acidification of the medium due to glutaric acid presence since when buffered to pH 7.4 no cytotoxic effects were observed (data not shown).

A stably transfected hNa_v_ 1.6 HEK cell line was used to evaluate the activity of glutaric acid over sodium currents. Na_v_ 1.6 is the voltage gated sodium channel most expressed at nodes of Ranvier but it is also observed in axons and dendrites^29^ Na_v_ 1.6 channels produce well-documented persistent and transient sodium currents that are important for axonal conduction. Electrophysiological recordings were obtained in an automated electrophysiological platform where compound addition is regulated by a microfluidic system controlling a fast and precise solution application and exchange^30^. Serial dilutions (0.0001–100 mM) of glutaric acid (buffered to pH 7.4 and non-buffered) were made in extracellular solution and added to Na_v_ 1.6 transfected cells and the effects of the compound over the peak amplitude measured. As was the case in cytotoxicity assays only non buffered glutaric acid produced a marked effect in I_Na_ with a complete blockage of the current indicating again that the observed response was simply a function of reducing pH and not glutaric acid specific (data not shown). Since glutarylcarnitine was also detected, the same assays were performed with this metabolite although no toxicity effects were previously reported for it^31^. As with glutaric acid case, glutarylcarnitine did not show any effect over sodium currents (data not shown).

**Figure 4:**
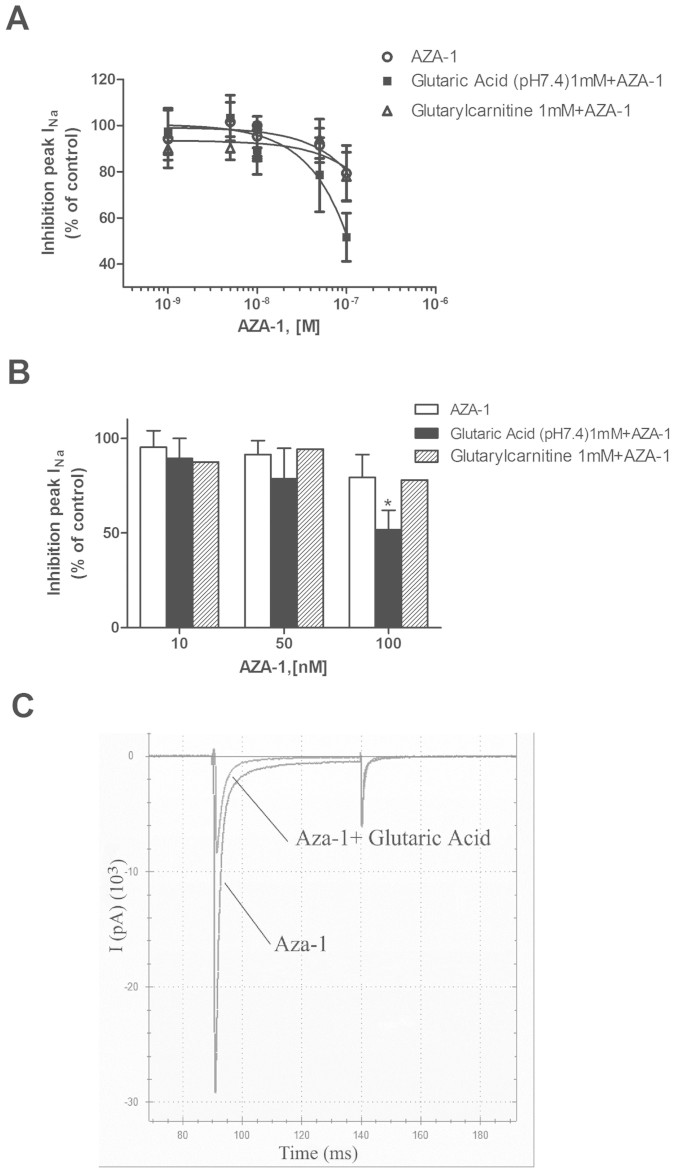
Effect of the co-application of glutaric acid or glutarylcarnitine over AZA-1 sodium currents modulation. (A) AZA-1 at nanomolar concentrations did not produce an inhibition of I_Na_ in Nav 1.6 transfected cells. However a decrease of the current was observed when AZA-1 was added to pretreated cells with 1mM glutaric acid. (B) Histogram showing the inhibition of I_Na_ peak with the highest AZA-1 concentrations tested in presence of glutaric acid or glutarylcarnitine. (C) Representative current traces of I_Na_ in the presence of 100 nM AZA-1 alone or in combination with glutaric acid (pH 7.4).

**Figure 5:**
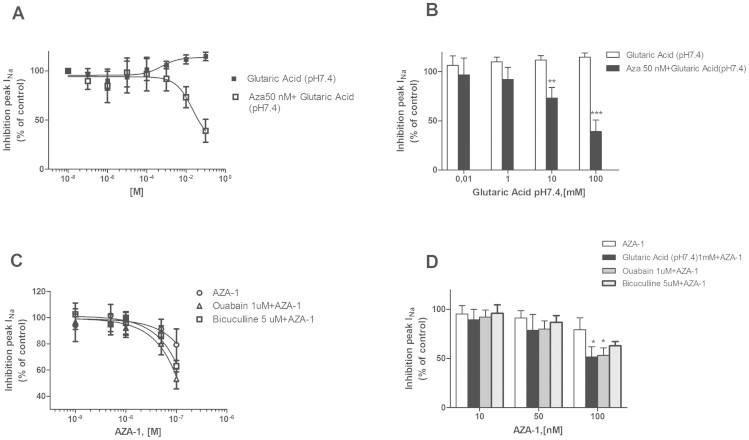
Effect of the co-application of AZA-1 over glutaric acid and glutarylcarnitine sodium current in Nav 1.6 stable transfected cells. (A) Glutaric acid in the mM range did not inhibit sodium currents. However the presence of AZA-1 50 nM enhanced its activity with a decrease of sodium currents at glutaric acid 10 and 100 mM. (B) Histogram showing the inhibition of I_Na_ peak with the highest concentrations of glutaric acid tested in presence of AZA-150 nM. (C) Effect of co-applications of ouabain (1 µM) or bicuculline (5 µM) over AZA-1 sodium currents modulation. The presence of Na^+^/K^+^ pump inhibitor ouabain produced an I_Na_ peak inhibition with the highest AZA-1 concentrations. No effect was observed with the GABA antagonist bicuculline. (D) Histogram showing the inhibition of I_Na_ peak with the highest AZA-1 concentration tested in presence of glutaric acid or ouabain or bicuculline.

The next phase of the study was to determine if the presence of glutaric acid or glutarylcarnitine can modify the effect of AZA-1 over I_Na_. Since glutaric acid produced a dose-dependent modification in pH, 1mM was selected for the experiment, a dose that did not affect the pH and that has been previously used in in vitro assays to study its effects in neurological systems^32^,^33^. Cells were incubated for 5 min with this proven nontoxic concentration (1mM) and nanomolar concentrations (1–100 nM) of AZA-1 which alone have been shown not to modify I_Na_ when added to the extracellular solution (Fig. 4A). These experiments showed that when 1mM glutaric acid (pH 7.4) was present in the extracellular solution a decrease of I_Na_ is observed with concentrations of AZA-1, ranging from 10 to 100 nM. The presence of 1mM buffered glutaric acid plus AZA-1 inhibited I_Na_ by 51.73% ± 12.72 (p = 0.03) (Fig. 4A and 4B).This data showed a glutaric acid specific effect when present in combination with AZA-1. However, the related compound detected in mussels, glutarylcarnitine (1 mM), did not affect the AZA-1 effects over I_Na_. To clarify the relationship between AZA-1 and glutaric acid further experiments were performed. Firstly, cells were incubated for 5 min with AZA-1 50 nM prior to addition of glutaric acid (pH 7.4) in concentrations ranging from 0.001 to 100 mM. These concentrations had been previously shown not to modify I_Na_, but surprisingly after AZA-1 incubation a dose dependent effect can be observed where 10 mM glutaric acid (7.4 mM) inhibited I_Na_ by 26.82 ± 10.6% and 100 mM by 60.97 ± 11.68% (p = 0.019 and 0.001 respectively)(Fig. 5A and 5B). However, when AZA-1 (50 nM) was added at the same time with each glutaric acid concentration no effect is observed, indicating that a preliminary activation of some target may be necessary (data not shown).

Since glutaric acid has showed Na+/K+-ATPase activity^34^, an established inhibitor of this pump (ouabain) was used to verify if the observed effect could be mediated by the effect of glutaric acid over it. Cells were incubated for 5 min with 1 µM ouabain prior to the addition of AZA-1. Once again a similar effect was observed, where the highest concentrations tested produced a clear inhibition of I_Na_ in the presence of ouabain inhibiting the current in a 46.48 ± 9.25% (p = 0.03) (Fig. 5C and D). Moreover, glutaric acid neurotoxicity has also been related to an impairment of gamma-aminobutyric acid (GABA) production and a consequent decrease in its levels^35^, thus the effects of this GABA imbalance over AZA-1 effects were also tested. The GABA antagonist bicuculline (5 µM) was also added to a cellular solution for 5 min before AZA-1 application. No significant effect of AZA-1 inhibition of sodium currents was found. These results strongly suggest that the effect of glutaric acid over the Na^+^/K^+^-ATPase was responsible for the observed effects and that the combination of both substances is required to cause the observed effect on sodium channels.

## Discussion

The symptoms of azaspiracid poisoning have never been explained satisfactorily in terms of describing the target for the toxin. The data presented provide a plausible explanation of why neurological effects are observed after extracts containing AZA-1 or partially purified AZA-1, which contain glutaric acid, affect voltage-gated sodium ion channels. Previously, it has been shown that AZA-1 produces an irreversible inhibition of the bioelectrical activity in spinal cord neuronal networks but it failed to show any effect over sodium or calcium voltage-gated channels^36^. However AZA-1 was only tested in concentrations up to 10 nM and purified AZA-1 (> 93%) was used to perform all the assays. In this current study it has been demonstrated that concentrations <100 nM of AZA-1 have no effect on sodium currents, yet a significant effect was observed in the presence of 1 mM glutaric acid. The levels of glutaric acid found in the shellfish were in the low µg/mL range and that used in the toxicity studies was a medium µg/mL dose. Further studies to determine the amount of glutaric acid required to cause the synergistic effect with AZA are required.

Azaspiracid poisoning is a fairly new phenomenon. The first evidence of the toxin appeared less than 20 years ago, it suddenly emerged on the West coast of Ireland and has continued to do so episodically over this period remains a mystery. The toxin has been found in many other geographic locations but the only confirmed cases of associated illness from consuming contaminated shellfish are linked with shellfish harvested from Irish waters. It may be the coming together of the azaspiracid toxins, glutaric acid and perhaps as yet other unknown compounds in filter feeding shellfish are required to cause the toxic consequences in humans. Changes in oceans currents caused by climate change may be another attributing factor that has been responsible for the bringing together of all the ‘ingredients’ needed to form a cocktail of chemicals in shellfish that can result in severe food poisoning. It is interesting to note that AZA has now been detected in shellfish harvested in many parts so the world^8^,^9^,^10^ but toxicity has only been associated with product harvested in west, south west and north westerly Irish waters. It is possible that the toxin has been present in shellfish from this region for a considerable time period and the ‘change’ has been the introduction of a source of glutaric acid and possibly other toxic dicarboxylic acids. It has been shown that the occurrence of glutaric acid is strongly influenced by marine, biogenic emissions^37^. Co-incidentally but perhaps not a recent study published by Grenfell^38^ presented data to show greatly enhanced biogenic emissions were recorded (1996–1997) at a remote coastal site in western Ireland where many azaspiracid outbreaks have occurred and at a time close to when the first azaspiracid outbreaks was recorded (1995). The finding of glutaric acid in all shellfish samples analysed (n = 10) from this coastal region strongly supports this report however a larger scale study of shellfish from a variety of coastal regions is required to build the level of knowledge on the possible role of biogenic emissions and toxin accumulation is filter feeders.

The hypothesis tested in this study was further supported by the potentiatory effect of glutaric acid on the toxic response to low levels of azaspiracid. Glutaric acid has been very recently reported to have an apoptotic effect in neurons^39^, targeting similar cellular structures to azaspiracids such as mitochondria and cell morphology^19^. A combined neurotoxicity of azaspiracid with glutaric acid has been demonstrated for the first time. The fact that both compounds were found to be present in shellfish harvested from an area associated with food poisoning episodes that manifested neurological effects may well provide an explanation for such events.

## Methods

### LC-MS/MS analysis of shellfish samples

The EU reference laboratory (EU-RL) liquid chromatography mass spectrometry method (LC-MS/MS) method (http://www.aesan.msps.es/en/CRLMB/web/home. shtml) was utilised as the reference method (Regulation EC 15/2011) for the determination of lipophilic toxins including azaspiracids 1,2,3 as a requirement of the Irish monitoring programme. The total azaspiracid content was calculated as azaspiracid equivalents to AZA-1. The toxin content of whole mussel (*Mytilus edulis*) samples was determined to select a collection of samples over a time period from one location whereby both non-contaminated and contaminated samples with azaspiracid could be sourced without the presence of other regulated marine toxins.

All solvents (methanol, acetonitrile, formic acid) were purchased from Sigma-Aldrich (Dorset, UK) and were LC-MS grade or equivalent. Ultra-pure water (18.2 MΩ·cm^−1^) was generated in-house using a Millipore Milli-Q Integral (Merk Millipore, Billerica, MA, USA) water purification system. Plastic tissue-culture dishes were purchased from Falcon (Madrid, Spain). Foetal calf serum, Dulbeccós modified Eagle medium/F12 nutrient mixture (DMED/F12), Gluatamax, Minimum essential medium, non-essential amino acids (MEM NEAA) and G418 were purchased from Gibco (Glasgow, UK). Detachin™ was purchased from Genlantis (USA). Glutaric Acid and all other chemicals (of reagent grade) were purchased from Sigma-Aldrich (UK). Azaspiracid-1 standard (99.9% of purity) was from Laboratorio Cifga (Lugo, Spain).

#### **Preparation of mussel samples**

Mussel samples were collected and flash frozen in liquid nitrogen for storage at −80°C. Frozen samples were lyophilized (Christ 4L, IMA Life Sciences, US) and milled to a fine powder under liquid nitrogen (Freezermill, SpexSamplePrep, USA). Dried homogenate (0.05g) was extracted in 1 mL of methanol/water solution (1:1, v/v), mixed for 30 minutes, centrifuged at 4,000 *g* for 10 minutes at 4°C and the supernatant collected. The supernatant was dried and reconstituted in 700 µL of ultra-pure water. Subsequently, the extract was filtered through a 0.22 µm Costar Spin-X Centrifuge Tube Filter (10,000 *g* at 4°C for 5 minutes). Filtered extracts were immediately transferred into Waters Maximum recovery vials for UPLC-QTof-MS analysis. A pooled sample (QC) was prepared from 30 µL of extract from each vial.

#### **UPLC-QTof-MS analysis**

Chromatography was performed on a Waters Acquity UPLC I-Class system(Milford, MA, USA), equipped with column oven, coupled to a Waters Xevo G2-S QTof mass spectrometer (Manchester, UK) equipped with an electrospray ionisation source operating in positive mode with lock-spray interface for real time accurate mass correction. The source temperature was 120°C with a cone gas flow of 50 L/h, a desolvation temperature of 450°C, and a desolvation gas flow of 850 L/h. The capillary voltage was set at 1.0 kV with a cone voltage of 30 V. Source offset was 60 (arbitrary unit). Mass spectra data were acquired in centroid mode using MSE function (low energy: 4eV; high energy: ramp from 15 to 30 eV) over the range m/z 50–1200 with a scan time of 0.1s. A lock-mass solution of Leucine Enkephalin (1 ng.µL^−1^) in acetonitrile/water containing 0.1% formic acid (1:1, v/v) was continuously infused into the MS via the lock-spray at a flow rate of 5 µL.min^−1^.

A 1.5 µL aliquot of extracted shellfish sample was injected onto an Acquity UPLC HSS T3 column (2.1 × 100 mm, 1.8 µm, Waters, Milford, MA, USA). The column oven was set at 45°C, and the sample manager temperature was 6°C. The gradient elution buffers were A (water with 0.1% formic acid) and B (methanol with 0.1% formic acid), and the flow rate was set at 0.4 mL.min^−1^. The elution gradient (A:B, v/v) was as follows: an isocratic period of 2 min at 99.9:0.1 followed by a concave gradient from initial conditions to 1:99 over 19 min. After a 2 min isocratic period at 1:99, a linear gradient was applied over 0.1 min to return to the initial composition 99.9:0.1 which was held for 1.9 min before the next injection.

Prior to all analyses 10 pooled conditioning samples were injected. For quality control pooled samples were injected at intervals every 10 samples throughout the entire experiment to determine the chromatographic reproducibility of retention times and peak intensities^25^.

#### **UPLC-MS/MS analysis**

Glutaric acid measurement was carried out using a Waters Acquity UPLC I-Class coupled to a Waters Xevo TQMS triple quadripole mass spectrometer (Manchester, UK) operating in negative electrospray ionisation. The source temperature was 150°C with a cone gas flow of 50 L/h, a desolvation temperature of 400°C, and a desolvation gas flow of 700 L/h. The capillary voltage was set at 2.0 kV with a cone voltage of 20 V. Data acquisition was in multiple reaction monitoring mode (MRM). The precursor/product ions monitored were 131.00> 68.95 (collision energy 15 eV), 131.00> 86.95 (collision energy 10 eV) and 131.00> 112.95 (collision energy 10 eV). A 5.0 µL aliquot of extracted shellfish sample was injected onto an Acquity UPLC BEH column (2.1 × 50 mm, 1.7 µm, Waters, Milford, MA, USA) maintained at 45°C. Mobile phase consisted of water with 0.1% formic in channel A and methanol with 0.1% formic acid in B. The flow rate was set at 0.4 mL.min^−1^. The gradient profile was isocratic (99:1) for 1.8 min then linear to 1:99 over 0.1 min. These conditions were maintained for 1.1 min and then followed by a 2 min re-equilibration period at initial conditions (99:1). Data was processed using TargetLynx™ software (Waters, Milford, US).

#### **Data analysis**

The raw data from the spectral analysis of the mussel extracts was processed using TransOmics v1.0 Software (Waters Corporation, Milford, USA). TransOmics is a new data analysis software package that enables the accurate processing of high resolution LC-MS spectral data whilst also providing annotation of putative compounds based on accurate mass measurements and fragmentation information. This software aids in both the validation of LC-MS approaches and identification of features within the spectral data. Using TransOmics the spectral data were aligned to a chosen pooled sample, adduct ions were deconvoluted and ions abundance of features above the threshold level calculated. All detected ions were selected against the Progenesis Metascope “Biomolecules” database which provided putative identifications for around 20% of the identified features.

Following analysis in TransOmics, the raw data were filtered to remove any variables containing >20% zero values (considered technical noise). The data were exported to Simca v13.0.3 (Umetrics, Umea, Sweden) for multivariate analysis. As a quality control measure all the spectral data were Center Scaled and analysed using principal components analysis (PCA). All pooled samples were found to be tightly clustered within the center of each representative scores plot which indicates good reproducibility of the data^25^. Following this, all data were mean centered and Pareto scaled (scaling factor 1/√sd) and divided into two groups: toxin negative and toxin positive prior to analysis by Orthogonal Projections to Latent Structures-Discriminant Analysis via Partial Least Squares (OPLS-DA). This method was employed as it ensures the inclusion of metabolites present in both high and low levels in the model, while minimizing the effect of noise^40^. Predictive powers were based on the Q2 score produced using Simca P. Essentially the data are divided into 7 parts (by default) and each 1/7th in turn is removed. A model is built on the 6/7th data left in and the left out data are predicted from the new model. This is repeated with each 1/7th of the data until all the data have been predicted. The predicted data are then compared with the original data and the Predicted Residual Sum of Squares (PRESS) is calculated for the whole dataset. Q2 is calculated form the PRESS value which is divided by the initial sum of squares and subtracted from one. Models with good predictive powers have low PRESS scores and high Q2 values^25^,^41^. Graphical representations of the data were produced using Prism (Version 5.0).

The ions of interest (highlighted by the S-plot) at different levels between the two sample groups were analysed using a two tailed homoscedastic student t test (assuming that the two sample groups were of equal variance) using Microsoft Excel (2010)^25^.

### Cell culture

Neuroblastoma cell line SH-SY5Y was purchased from ATCC. Cells were plated in 25 cm^2^ flasks and maintained in DMEM/F12 media supplemented with 10% foetal serum, 100 UI.mL^−1^ penicillin and 100 µg.mL^−1^ streptomycin. Cells were split weekly using 0.05% trypsin/EDTA.

HEK-293 cell line stably transfected with hNa_v_ 1.6 was kindly provided by Dr Andrew Powell (GlaxoSmithKline R&D, Stevenage, UK). Cells were cultured in DMEM/F12 medium supplemented with Glutamax, MEM NEAA (1% w/v) and 10% of foetal bovine serum. G418, 0.4 mg.mL^−1^ was added to the medium. Cells were incubated in a humidified 5% CO_2_/95% air atmosphere at 37°C until they reached 80% of confluence. Medium was replaced every 2–3 days and split once a week. Cells with 80% of confluence are incubated at 30°C for 24–48 h before electrophysiological measurements.

### Cytotoxicity assays

Cell viability was assessed by a MTT (3-[4,5-dimethylthiazol-2-yl]-2,5-diphenyltetrazoliumbromide) test as previously described^42^. Cells were grown in 96 well plates and incubated with ranging concentrations of the tested compound (from 0.0001–100 mM) in culture medium. Cultures were maintained in the presence of the compound at 37°C in humidified 5% CO_2_/95% air atmosphere for 24 or 1 hour after the exposure time, cells were rinsed and incubated for 60 min with a solution of MTT (500 µg.mL^−1^) dissolved in Locke's buffer containing (in mM): 154 NaCl, 5.6 KCl, 1.3 CaCl_2_, 1 MgCl_2_, 5.6 glucose and 10 HEPES, pH 7.4 adjusted with Tris. After washing off excess MTT the cells were disaggregated with 5% sodium dodecyl sulphate and absorbance of the colored formazan salt was measured at 590 nM in a spectrophotometer plate reader. Saponin was used as a cellular death control and its absorbance values were subtracted from the other data.

### Automated patch clamp electrophysiological recordings

All cells were recorded in whole-cell patch clamp configuration using an IonFlux 16 system (Fluxion, California, USA) and the corresponding Ionflux 16 software for cell capturate, seal formation, whole cell obtaining, data acquisition and analysis. Cells maintained at 30°C for 24–48 h were washed twice with Ca^2+^ and Mg^2+^ free phosphate buffered saline (PBS) and harvested with 5 mL of Detachin™ solution. After cell detachment, cells were re-suspended in extracellular solution containing (mM): 2 CaCl_2_, 1 MgCl_2_, 100 Hepes, 4 KCl, 145 NaCl, 10 TEA-Cl and 10 Glucose. pH 7.4 and 320 mOsm. Electrophysiological recordings were carried out at room temperature (± 22°C) in a 96-well IonFlux microfluidic plate. This system consists of an automated patch clamp platform based in a microfluidic system where cells in suspension are captured by suction into ensemble recordings arrays formed by 20 individual micro-channels for cell voltage clamp in parallel, each of which will trap one cell. Once that the recording assay is full, suction is applied to obtain whole cell configuration by cellular membrane breakage^30^.

For Na^+^ measurements in hNav1.6 channels, sodium currents (I_Na_) were evoked by the following protocol. Cells were depolarized to −10 mV for 50 ms after a 100 ms step to −120 mV from −90 mV holding potential (V_h_). The intracellular solution composition for I_Na_ recordings was (in mM): 100 CsF, 45 CsCl, 10 Hepes, 5 NaCl, 5 EGTA corrected to pH 7.4 using CsOH. The contaminating effects of resistance and capacitance currents were compensated electronically by the software. Leak resistance is measured by introducing a short 20 mV pulse at the beginning of each sweep and measuring the current difference^30^. A sampling frequency of 10 kHz was used.

### Statistical analysis

Data analysis was performed with GraphPad Prism 5 software. All the assays were performed at least three times. Dose-response curves were analysed using nonlinear regression. Statistical comparison was performed by Student's t-test. *P* values <0.05 were considered as statistically significant. All data are shown as the means ± SEM.

## Author Contributions

E.C.T. and B.L.M. designed the experiment, supervised the project and co-wrote the manuscript. C.K. designed the experiment. D.C. and S.J. performed the experiment on targeted analysis (AZA measurement) and co-wrote the manuscript, C.O.P. carried out untargeted and targeted analysis (glutaric acid measurement), data analysis and co-wrote the manuscript. G.S.F. carried out data analysis and co-wrote the manuscript. A.E. carried out the toxicology study and co-wrote the manuscript.

## Figures and Tables

**Table 1:** Glutaric acid concentrations measured in extracts of blue mussel (Mytilus edulis) by LC-MS/MS

Sample type	Glutaric acid conc (µg.g^−1^)
AZA positive 1	51.49
AZA positive 2	40.78
AZA positive 3	29.50
AZA positive 4	56.21
AZA negative 1	33.15
AZA negative 2	51.27
AZA negative 3	81.10
AZA negative 4	34.99
Commercial 1	62.50
Commercial 2	48.56

**Table 2:** A list of the ions found to be at significantly higher levels (p <0.05) in AZA samples (Pool 2) when compared to controls (Pool 1) as measured in ESI+ and as depicted in Fig. 3

		RT	M/Z	Putative ID	Azaspiracid (n = 56)	SEM	Controls (n = 92)	SEM	p-Value
**ESI+**	**1**	1.22	216.1237	Propenoylcarnitine	1.70E+06	3.01E+04	1.08E+06	6.25E+04	8.92E-12
	**2**	1.22	262.1293	N/A	2.32E+07	4.07E+05	1.45E+07	8.47E+05	2.39E-12
	**3**	1.23	280.1395	N/A	7.12E+06	1.16E+05	4.39E+06	2.44E+05	5.00E-14
	**4**	1.53	294.1010	N/A	3.15E+06	1.19E+05	1.24E+06	8.20E+04	9.11E-28
	**5**	1.72	136.0761	2-Aminoacetophenone	5.12E+06	1.94E+05	4.08E+06	1.32E+05	1.03E-05
	**6**	1.86	1122.4299	N/A	1.86E+06	5.32E+04	7.01E+05	7.12E+04	3.23E-22
	**7**	2.11	326.1235	N/A	7.46E+06	2.29E+05	3.00E+06	2.11E+05	3.28E-28
	**8**	2.15	344.1338	N/A	5.35E+06	1.53E+05	2.21E+06	1.51E+05	2.07E-28
	**9**	3.06	276.1448	Glutarylcarnitine	5.20E+07	1.43E+06	3.27E+07	2.20E+06	2.34E-09
	**10**	3.08	258.1338	N/A	4.25E+06	1.19E+05	2.74E+06	1.75E+05	5.03E-09
	**11**	3.08	294.1551	N/A	2.18E+07	5.27E+05	1.37E+07	8.85E+05	3.08E-10
	**12**	3.10	230.1392	Butenylcarnitine	2.74E+06	6.87E+04	1.74E+06	1.13E+05	1.19E-09
	**13**	3.24	285.1149	N/A	2.55E+06	6.96E+04	1.68E+06	7.29E+04	3.54E-13
	**14**	4.60	120.0814	N/A	2.74E+07	1.72E+06	2.26E+07	5.78E+05	2.17E-03
	**15**	4.61	166.0867	Phenylalanine	5.90E+06	2.04E+05	4.61E+06	1.35E+05	1.73E-07
	**16**	6.40	310.1288	N/A	1.52E+07	7.92E+05	7.60E+06	5.05E+05	2.32E-14
	**17**	6.40	328.1389	N/A	7.52E+06	3.67E+05	3.86E+06	2.51E+05	2.03E-14
	**18**	6.43	292.1183	N/A	1.15E+06	5.71E+04	6.05E+05	3.76E+04	8.05E-14
	**19**	6.53	285.1457	N/A	3.52E+07	9.74E+05	3.09E+07	7.15E+05	4.52E-04
	**20**	7.14	780.3134	N/A	9.61E+05	4.39E+04	3.65E+05	4.13E+04	7.77E-17
	**21**	7.93	188.0716	N/A	3.89E+07	1.52E+06	3.00E+07	6.81E+05	1.43E-08
	**22**	7.95	118.0655	N/A	2.52E+06	1.35E+05	1.81E+06	4.45E+04	2.08E-08
	**23**	7.95	146.0605	1H-Indole-3-carboxaldehyde	6.38E+06	3.52E+05	4.52E+06	1.24E+05	2.84E-08
	**24**	7.96	144.0811	N/A	4.40E+06	1.61E+05	3.48E+06	5.81E+04	2.67E-09
	**25**	7.96	205.0978	Tryptophan	3.14E+06	5.51E+04	2.47E+06	4.40E+04	8.44E-17
	**26**	8.24	377.1918	N/A	2.37E+06	3.85E+05	7.22E+05	8.43E+04	7.30E-07
	**27**	8.53	349.1391	N/A	2.32E+06	7.45E+04	1.04E+06	7.23E+04	1.36E-22
	**28**	8.53	367.1498	N/A	4.28E+06	1.35E+05	1.86E+06	1.36E+05	3.82E-23
	**AZA**	20.07	842.5049	Azaspiracid	2.67E+05	3.03E+04	8.35E+02	1.27E+02	1.24E-21

## Notes

All authors reviewed the manuscript.
